# Quality control system for mammographic breast positioning using deep learning

**DOI:** 10.1038/s41598-023-34380-9

**Published:** 2023-05-01

**Authors:** Haruyuki Watanabe, Saeko Hayashi, Yohan Kondo, Eri Matsuyama, Norio Hayashi, Toshihiro Ogura, Masayuki Shimosegawa

**Affiliations:** 1 School of Radiological Technology, Gunma Prefectural College of Health Sciences, Maebashi, Japan; 2 Department of Radiology, National Hospital Organization Shibukawa Medical Center, Shibukawa, Japan; 3 Graduate School of Health Sciences, Niigata University, Niigata, Japan; 4 Faculty of Informatics, The University of Fukuchiyama, Fukuchiyama, Japan

**Keywords:** Health care, Health occupations

## Abstract

This study proposes a deep convolutional neural network (DCNN) classification for the quality control and validation of breast positioning criteria in mammography. A total of 1631 mediolateral oblique mammographic views were collected from an open database. We designed two main steps for mammographic verification: automated detection of the positioning part and classification of three scales that determine the positioning quality using DCNNs. After acquiring labeled mammograms with three scales visually evaluated based on guidelines, the first step was automatically detecting the region of interest of the subject part by image processing. The next step was classifying mammographic positioning accuracy into three scales using four representative DCNNs. The experimental results showed that the DCNN model achieved the best positioning classification accuracy of 0.7836 using VGG16 in the inframammary fold and a classification accuracy of 0.7278 using Xception in the nipple profile. Furthermore, using the softmax function, the breast positioning criteria could be evaluated quantitatively by presenting the predicted value, which is the probability of determining positioning accuracy. The proposed method can be quantitatively evaluated without the need for an individual qualitative evaluation and has the potential to improve the quality control and validation of breast positioning criteria in mammography.

Breast cancer is the most common cancer globally, and reducing its mortality rate requires early detection^1,2^. Mammography is beneficial for early breast cancer diagnosis and reduces cancer mortality^3–5^. There is evidence of a decrease in the mortality rate by approximately 20% using mammography in breast cancer screening^6^. To achieve an appropriate breast cancer diagnosis, it is essential to ensure technical quality in mammography and provide suitable diagnostic imaging. The optimal image contrast of breast tissue in a mammography image is necessary for detecting abnormalities. Mammography guidelines^7,8^ define the imaging equipment, quality control, and imaging techniques necessary for proper mammography^9–11^.

Inappropriate breast positioning has been reported as the most common cause of mammographic imaging failure^12,13^. Mammographic positioning must be performed with a firm grasp of the essential theory. High-accuracy positioning requires the training of individual radiological technologists, and the acquisition and teaching of techniques are labor-intensive and difficult. Inappropriate positioning cannot be supplemented regardless of the availability of high-performance imaging equipment. Breast positioning is difficult due to the subjective evaluation of mammography using visual inspection. The acceptance criteria for the positioning of images are required to include all advanced mammary glands. These criteria systems^7,8^ have commonly used a system with four scales—perfect, good, moderate, inadequate (PGMI)—or a system with three scales—excellent, adequate, repeat (EAR)—which many countries have adopted. In Japan, a three-scale evaluation similar to the EAR system has been used^14^. However, the criteria in the guidelines are limited to three or four scales of visual evaluation. Visual and qualitative assessments determine mammographic propriety; therefore, there is considerable variability in visual assessment between individuals, and accuracy remains an issue.

In recent years, artificial intelligence related to computer vision has been used in the medical field^15,16^. Machine learning systems, particularly deep learning systems, have been used in mammography, mainly for classification and detection. Deep convolutional neural network (DCNN)-based learning has distinguished benign and malignant tumors^17,18^. Furthermore, DCNNs have been successfully used to detect breast density and tumors^19,20^. Deep learning has also been applied to positioning for X-ray examinations^21,22^. Although there are several approaches to assessing mammographic positioning using computer schemes^23^, to the best of our knowledge, there are no reports on the use of DCNN for the verification of optimal positioning. In this study, we propose a DCNN classification for the quality control and validation of positioning in mammography, in which each part of a mammogram can be detected automatically.

## Results

Table 1 shows the experimental results of classification accuracy that inframammary fold (IMF) and nipple images be divided into three scales (excellent, average, and poor) using DCNN models, including VGG16, Inception-v3, Xception, Inception-ResNet-v2, and EfficientNet-B0. The VGG16 model achieves the highest accuracy of 0.7836 and the highest recall, precision, and F1 score of 0.5807, 0.5864, and 0.5797, respectively. All networks except VGG16 showed an accuracy of about 0.74, slightly lower than VGG16. Comparing all the metrics of the DCNN models, recall, precision, and F1 score showed a tendency similar to that of accuracy. All values other than those of VGG and the precision of Inception-v3 and EfficientNet-B0 are less than 0.5. For the nipple, Xception achieves the best accuracy among all models, with an accuracy of 0.7278. EfficientNet-B0 records an accuracy of 0.7167, which is close to that of the best model, Xception. Inception-ResNet-v2 yields the lowest accuracy (0.5641). All values other than the precision of Inception-v3, Xception, and EfficientNet-B0 in the recall, precision, and F1 score are less than 0.5.

**Table 1:** Classification performance of DCNN models in IMF and nipple.

Positioning part	DCNN models	Accuracy	Recall	Precision	F1 score
IMF	VGG16	0.7836	0.5807	0.5864	0.5797
	Inception-v3	0.7345	0.4543	0.5157	0.4700
	Xception	0.7413	0.4158	0.4971	0.4273
	Inception-ResNet-v2	0.7351	0.3943	0.4473	0.3880
	EfficientNet-B0	0.7430	0.4061	0.5237	0.4180
Nipple	VGG16	0.6849	0.4466	0.4830	0.4568
	Inception-v3	0.6947	0.3853	0.5076	0.3866
	Xception	0.7278	0.3686	0.6839	0.3472
	Inception-ResNet-v2	0.5641	0.3674	0.3602	0.3479
	EfficientNet-B0	0.7167	0.4044	0.5132	0.4113

**Figure 1:**
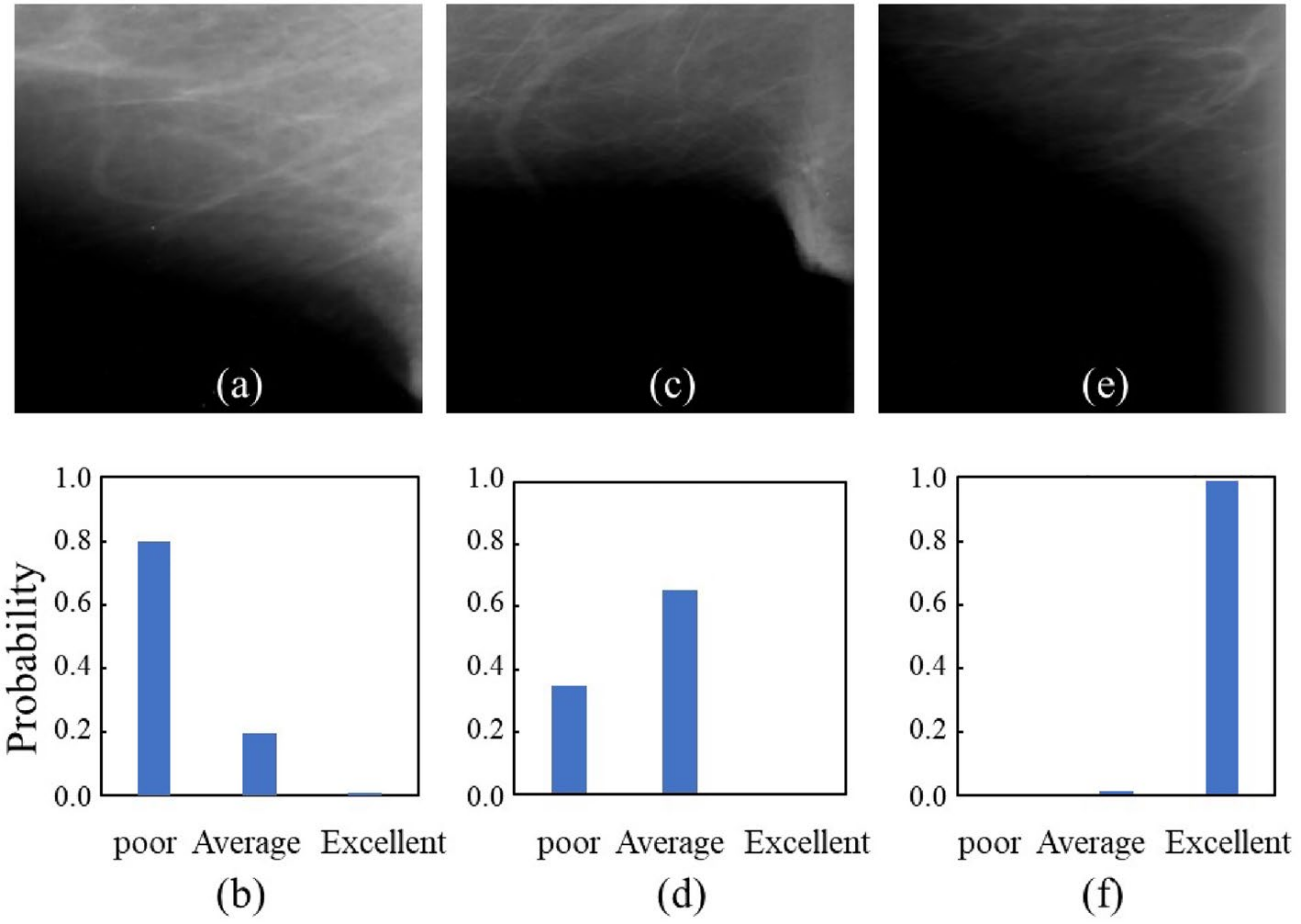
Correctly classified image examples with the probability of classification from the softmax function using VGG16 in the IMF. (**a**) Image labeled *poor*; (**b**) probability labeled *poor*; (**c**) image labeled *average*; (**d**) probability labeled *average*; (**e**) image labeled *excellent*; and (**f**) probability labeled *excellent* in being divided into three scales using DCNNs.

**Figure 2:**
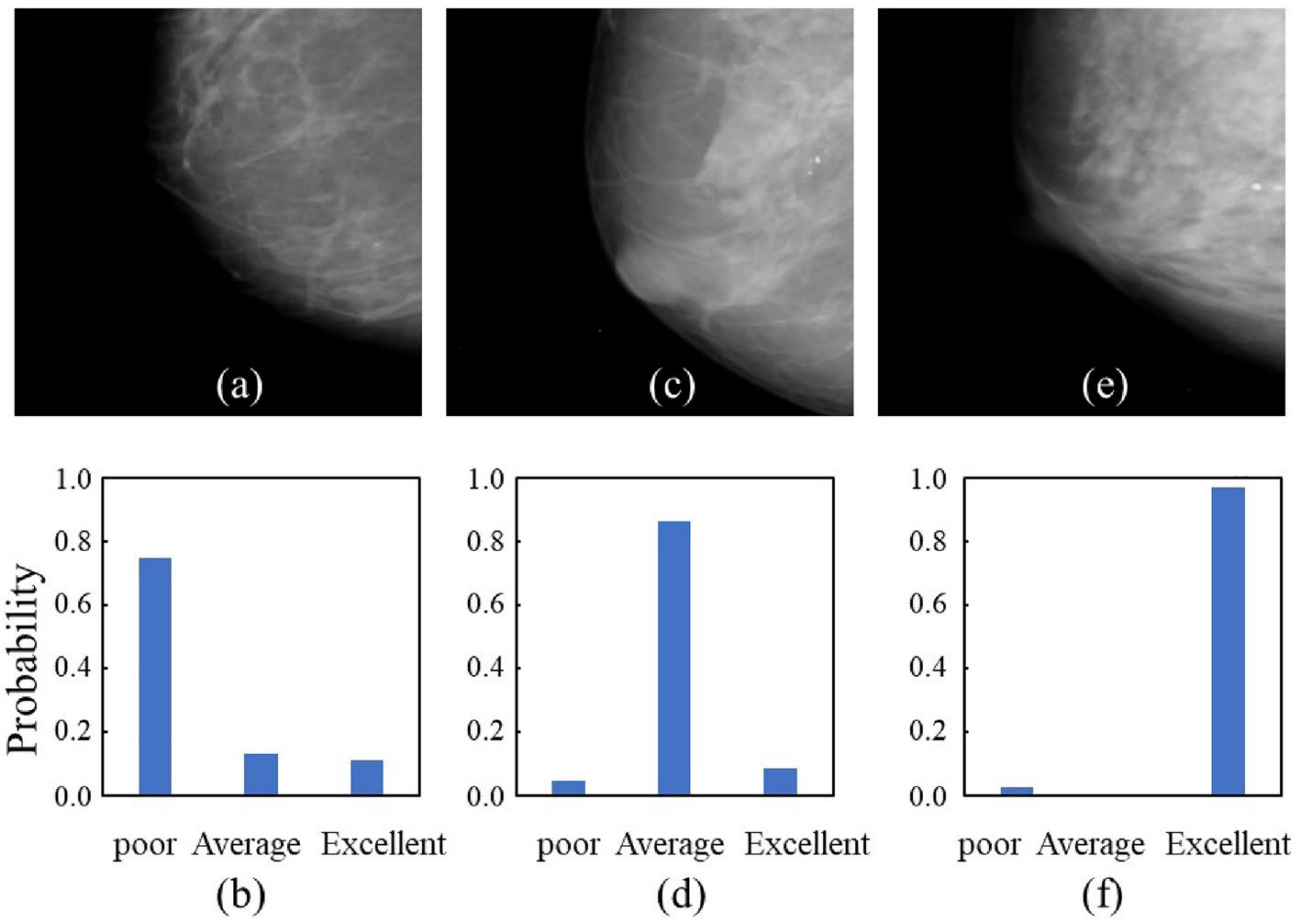
Correctly classified image examples with the probability of classification from the softmax function using VGG16 in the nipple. (**a**) Image labeled *poor*; (**b**) probability labeled *poor*; (**c**) image labeled *average*; (**d**) probability labeled *average*; (**e**) image labeled *excellent*; and (**f**) probability labeled *excellent* in being divided into three scales using DCNNs.

Figure 1 shows examples of correctly classified images of mammogram parts in addition to a probability calculated with the softmax function using VGG16 in IMF. In the IMF, the image labeled with poor, average, and excellent probability was 0.7971, 0.6547, and 0.9896, respectively. Figure 2 shows examples of correctly classified images of mammogram parts and a probability calculated with the softmax function using VGG16 in the nipple. In the nipple, the image labeled with poor, average, and excellent probability was 0.7514, 0.8656, and 0.9708 on the correct classified image, respectively.

## Discussion

In this study, we proposed that each part of a mammogram can be automatically detected, and breast positioning was classified based on quality control and validation using DCNNs. For the automatic classification of the IMF, the accuracy was 0.7836 (Table 1), the highest value obtained using VGG16. As an initial study, other metrics, such as recall, precision, and F1, were fairly useful (Table 1). The results demonstrated the feasibility of an automated evaluation of positioning techniques in mammography. The examples classified into three classes with a probability calculated with the softmax function are presented in Figs. 1 and 2. Softmax is a commonly used activation function in DCNN for image classification. The output of the fully connected layer is finally fed to the softmax function. The softmax function is a generalization of the logistic function, ensuring that our predictions add up to 1. Although the conventional visual classification in guidelines evaluated only three- or four-scale visual evaluation, the softmax function could obtain more detailed evaluation metrics, and the value predicted by the softmax function was between 0 and 1, including the decimal point, like a continuous value. By referring to these metrics, radiological technologists can determine the degree of their imaging technique and improve it.

The results of this study suggest that DCNNs can be used to classify mammographic breast positioning to evaluate imaging accuracy. The recognition of positioning criteria accuracy provides feedback to radiological technologists and can contribute to improving the accuracy of mammographic techniques. Our proposed method improves lesion detection performance because inaccurate imaging cannot capture subtle lesions. The softmax value output from the DCNN is a quantitative index, and by referring to that value, an even better mammogram accuracy can be evaluated. The disadvantages of exposure must always be considered in mammography. However, the judgment of re-taking that causes radiation exposure is left to the individual radiological technologists, and the basis for re-taking is poor and subjective. Therefore, it is crucial to guarantee imaging accuracy without relying on qualitative visual evaluation. This investigation indicates that quantitative evaluation could be made using the DCNN index without relying on individual subjectivity. This determines re-taking quantitatively and has the potential to reduce unnecessary medical exposures.

The nipple had a lower classification accuracy overall than the IMF (Table 1). Although Xception showed a moderate accuracy of 0.7278 (Table 1) owing to imbalanced data, the recall, and F1 score were less than 0.5; thus, it did not achieve classification performance. The learning image size seemed too large for the DCNNs to recognize nipple details. Improving accuracy requires adjusting the learning image size and localization. In addition, only grayscale processing using contrast limited adaptive histograph equalization (CLAHE)^24^ was performed in this study. Further improvement in accuracy is expected by performing other various grayscale corrections.

Regarding radiographic technique accuracy, previous work on image processing on the positioning of clinical imaging focused on the classification of anteroposterior and posteroanterior chest radiographs^21^. Further, an image evaluation method using a DCNN for skull X-ray images^22^ has been reported. The previous study of skull X-ray images attempted to classify positioning automatically but did not detect the target region of interest (ROI) automatically. A manual ROI setting is required in clinical applications, which is unrealistic. Our proposed method is useful because it includes the automatic detection of each part. The most recent attempts to evaluate mammographic breast criteria have not clarified the quantitative values derived from traditional image processing techniques^23^. We believe that our study is the first to address mammography breast positioning using DCNN classification. Our study showed that the automated detection of each part and appropriate DCNNs could help address breast positioning for quality control and assurance in mammography.

This study has several limitations. First, other positioning criteria, such as retro mammary space, pectoral muscle to nipple level, and symmetrical images for the image assessment of the mediolateral oblique (MLO) view in addition to the craniocaudal view, were not evaluated. Other parts should also be verified to establish a comprehensive evaluation. Second, we used an old database from a time when mammographic techniques had not been established, and the imbalanced data used had a considerable bias in the distribution of learning images. Further analysis and improvement in the performance of the DCNN model require additional research, including more recent and more extensive databases. Finally, the classification accuracy might have been influenced by the computer scheme, such as image size and localization, in the automated detection of learning and test images.

In conclusion, we focused on an issue that has not been addressed in previous studies using automated detection and DCNNs in mammographic breast positioning. The experimental results showed that the proposed method could be evaluated quantitatively without depending on individual qualitative evaluations, such as simply three- or four-visual scales. It can improve the quality control and validation of breast positioning criteria in mammography. As a future work, we will examine a quality control system using DCNNs on other positioning parts and improve the classification performance on the latest mammographic database.

## Materials and methods

**Figure 3:**
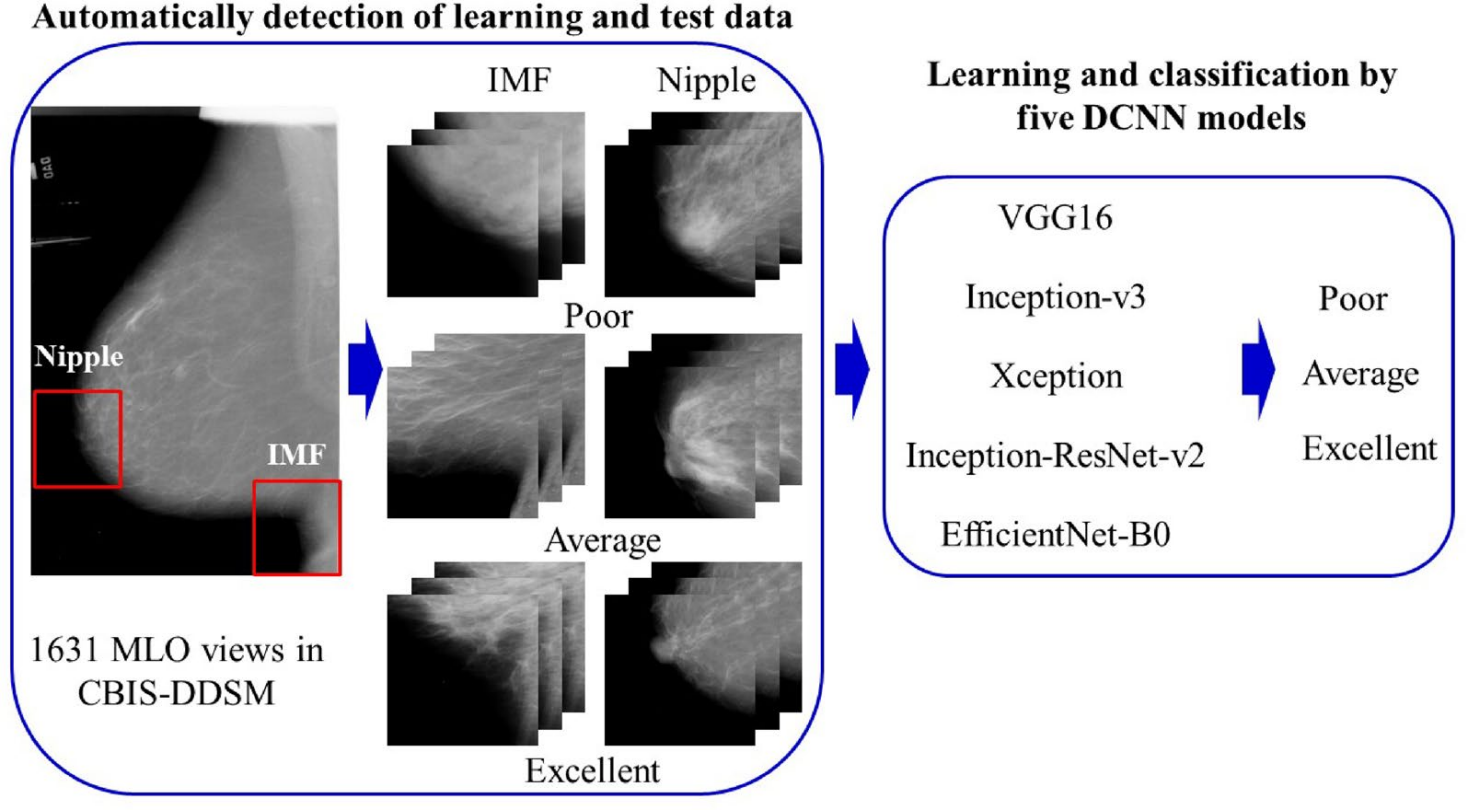
Working flow of the entire classification process divided into two steps: automated detection of the learning and test data and learning and classification by five DCNN models.

The entire mammography classification process proposed in this study is illustrated in Fig. 3. Labeled mammograms were correct images and were labeled by two radiological technologists specializing in mammography. The ROI in the labeled mammograph, which was visually evaluated in three classes (excellent, average, and poor), was extracted automatically from the IMF and nipple areas. If there was a disagreement in the three classes' evaluation, the evaluators discussed their selections until agreement was reached. The extracted ROIs were classified into three classes using five representative DCNN models. We obtained classification accuracy by comparing the labeled correct images and the DCNN classification results.

### Image dataset

The Curated Breast Imaging Subset of the Digital Database for Screening Mammography (CBIS-DDSM)^25^ is a large, publicly available dataset with open access for mammographic image analysis. The dataset consists of approximately 2600 scanned film mammography studies. We used only mediolateral oblique (MLO) views that consisted of 1631 images from the CBIS-DDSM, excluding extra annotations in mammography; this is because MLO is the basis of screening mammography and can detect a wide range of mammary glands. Mammography positioning is evaluated using guideline criteria in the pectoral major muscle, retro mammary space, bilateral breast symmetry, IMF, and nipple area. IMFs and nipples were targeted as a primary study.

Breast positioning assessment was performed by two radiological technologists engaged in screening mammography. Breast positioning criteria were selected from the three classes used in the Japanese guidelines^14^. The breast positioning criteria were visually classified into three classes (poor, adequate, and excellent) in IMF and nipple. Two radiological technologists reviewed the images and discussed the criteria to reach an agreement. A total of 1631 mammograms were labeled as ground truth. The IMF area images were classified into 1228 poor, 259 average, and 146 excellent images. The nipple area images were classified into 1169 poor, 275 average, and 189 excellent images.

Artificial intelligence classification requires training and testing of images. The automatic detection of target parts, such as the IMF and nipple, was performed on a Windows 10 personal computer with an NVIDIA GTX 2080Ti graphics processing unit and MATLAB software (version R2021a; MathWorks). Matching regions of the breast via the automated detection method on the mammographic image and identifying these regions by the classification method based on the DCNN achieved a satisfactory performance. In image preprocessing, mammograms of the left breast were flipped horizontally to the right. All mammographic images were resized to 2730 × 4096, and the intensity range was min–max normalized on a 0 to 255 scale in Portable Network Graphics (PNG) format.

### Automated detection of IMFs

**Figure 4:**
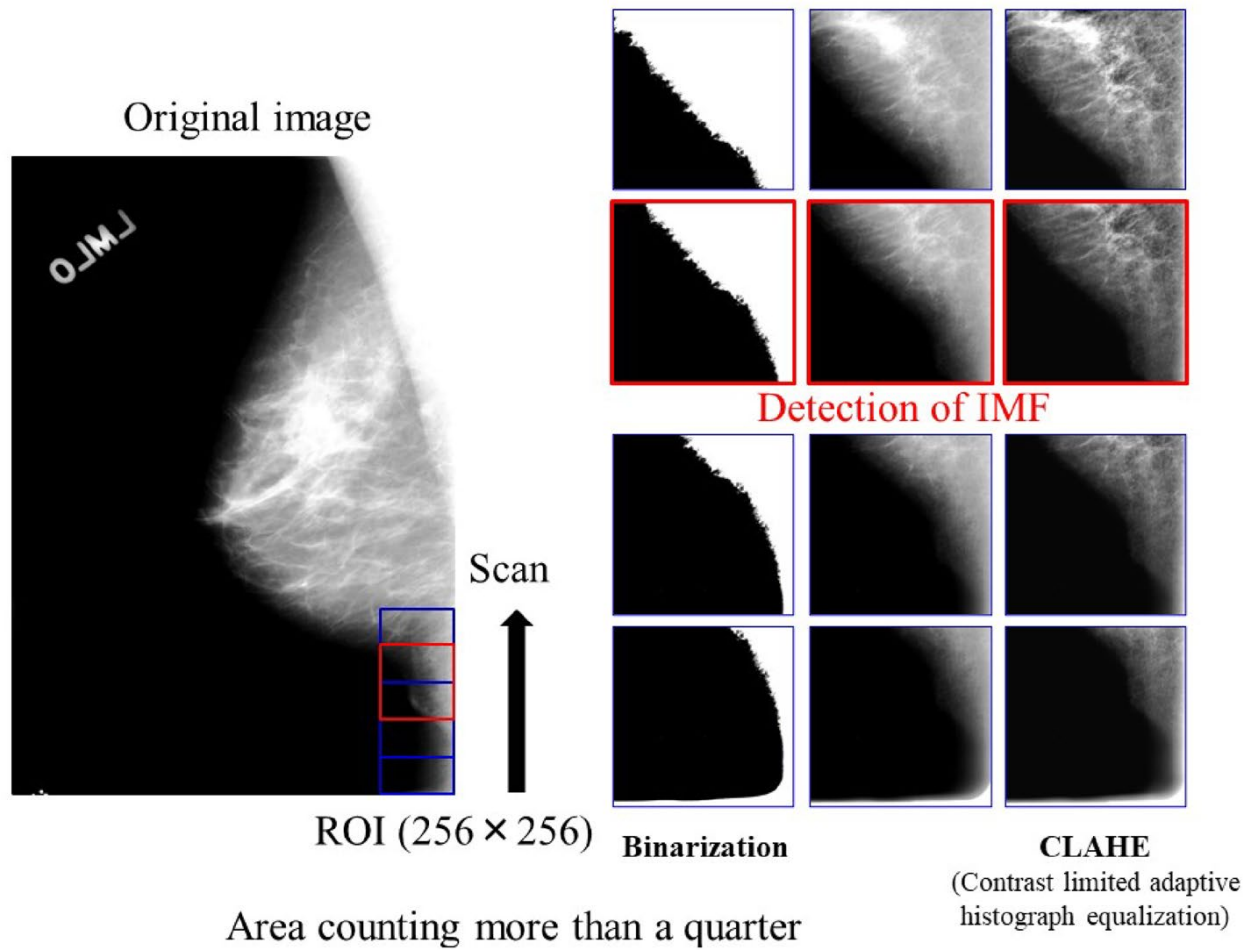
Automated detection of IMF scanned upward until the count of the number of pixels in the ROI was more than a quarter.

**Figure 5:**
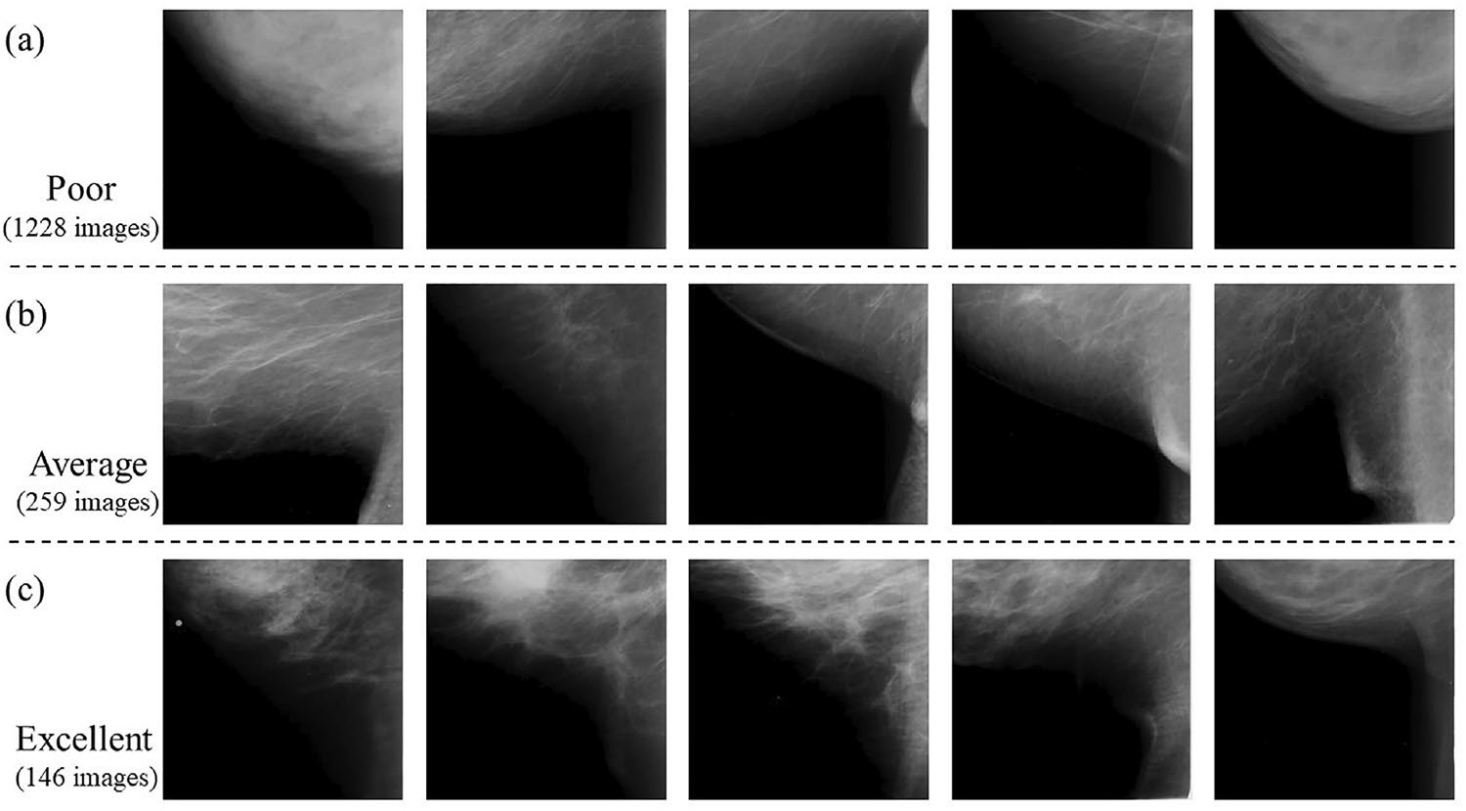
Examples of learning and test images classified into three classes: poor, average, and excellent images in IMF. (**a**) Images labeled *poor*; (**b**) images labeled *average*; and (**c**) images labeled *excellent*.

The processing flowchart of the automated detection of the IMFs is shown in Fig. 4. To detect the IMF, an ROI of 256 × 256 pixels was set at the bottom of the mammogram, and images in the ROI were converted to binary images using a global threshold (normalized value = 0.8). The number of pixels of IMF was counted in the ROI. If the number of pixels of IMF was less than a quarter of the number of pixels in the ROI, the ROI moved up in an image and counted the number of pixels again. If the number of pixels of IMF was more than a quarter of the number of pixels in the ROI, the ROI image was cropped as a final image. Finally, the image in the automatically generated ROI was cropped, and CLAHE, one of the grayscale processing methods, was applied and associated with the visual evaluation: 1. poor, 2. average, and 3. excellent. The extracted ROI, that is, the IMF, is shown in Fig. 5.

### Nipple in profile

**Figure 6:**
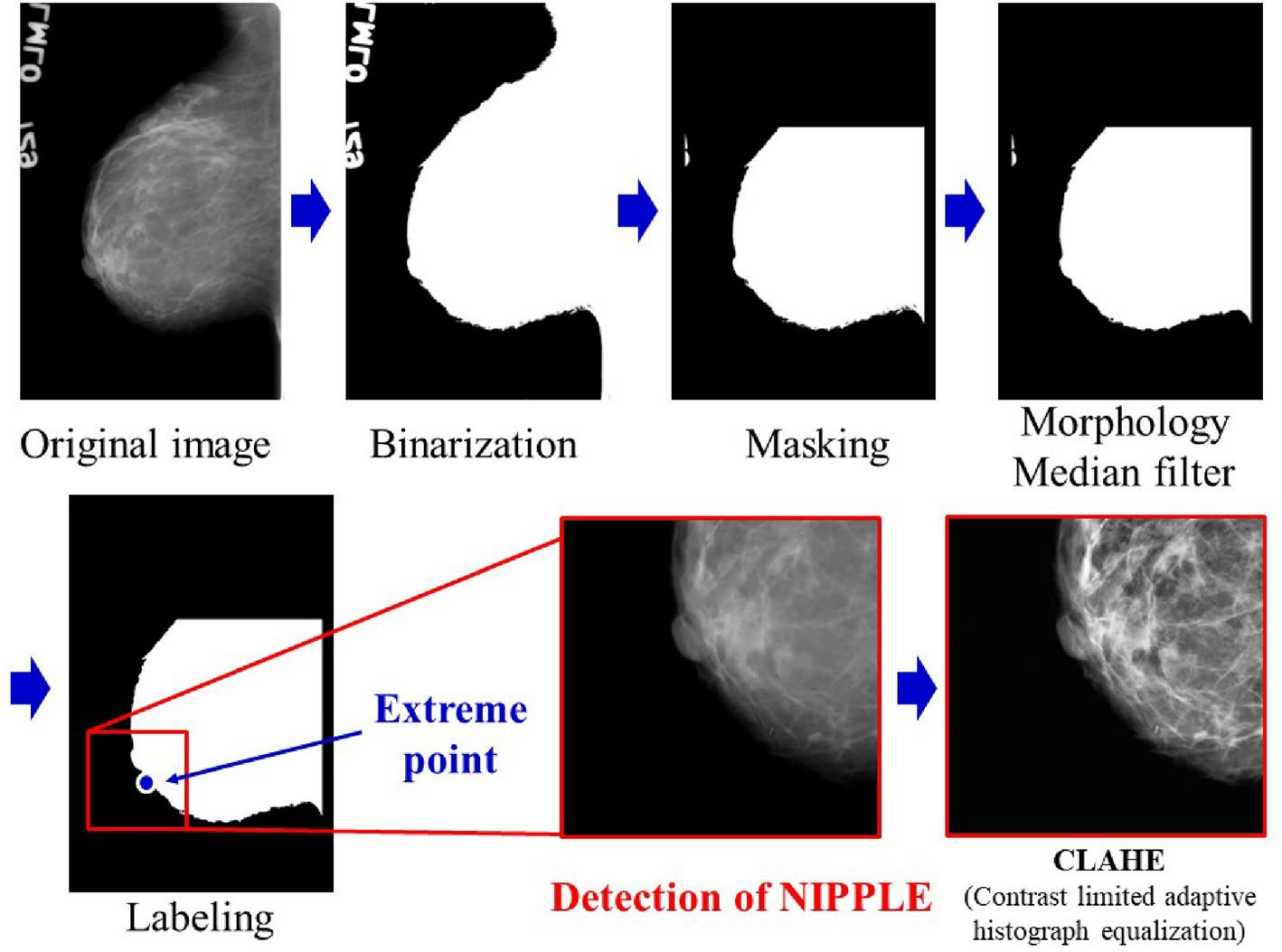
Automated detection of nipple. Extreme points near the nipple are captured by image processing, and the nipple area is detected.

**Figure 7:**
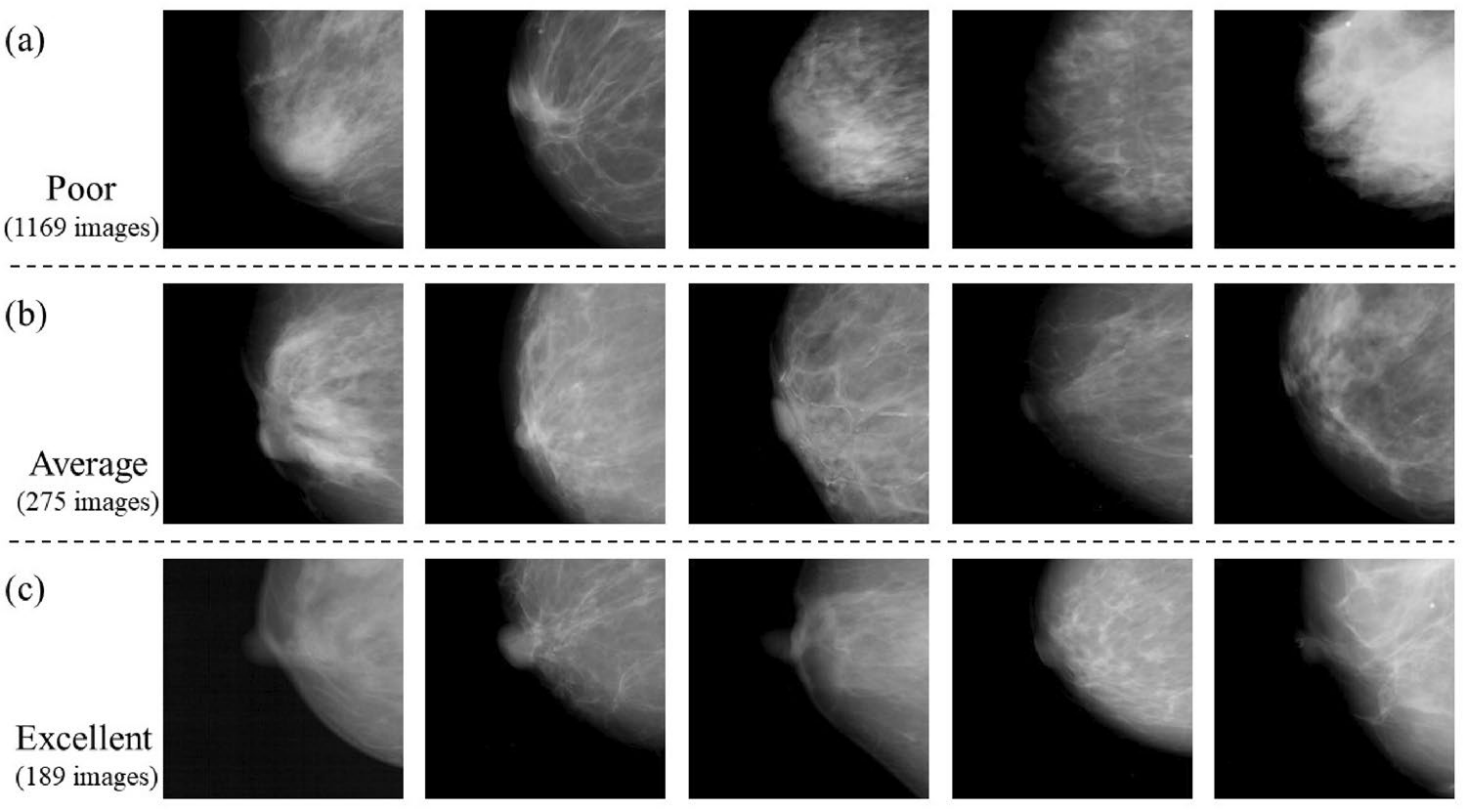
Examples of learning and test images of the nipple classified into three levels: poor, average, and excellent. (**a**) Images labeled *poor*; (**b**) images labeled *average*; and (**c**) image labeled *excellent*.

The processing flowchart of the automated detection of the nipple is shown in Fig. 6. The mammogram images were filtered using a median filtering approach in a 3 × 3 pixel neighborhood for denoising. After the grayscale mammogram images were converted to binary images, morphological filters were applied to the images to eliminate radiopaque artifacts and labels, such as orientation indicators. Most mammograms contain shading unrelated to the diagnosis on the top, bottom, or left–right sides. Hence, to remove the shading, binary masking was applied using a rectangular mask. Morphological operations, such as dilation, erosion, opening, and closing, were performed on the binary images. Pixels were counted, and the maximum number of pixels was used as the breast, and other parts were excluded using a labeling algorithm. The left-bottom extrema points and x- and y-coordinates of one of the points were detected using the measured properties of the breast regions^26^. An ROI with a size of 256 × 256 pixels was set around the extrema point as an index. The images in the ROI using the CLAHE were labeled through visual evaluation, similar to the IMF. The extracted ROI, which is the nipple in profile, is shown in Fig. 7.

### Deep convolutional neural network

In this study, four DCNN architectures, VGG-16^27^, Inception-v3^28^, Xception^29^, Inception-ResNet-v2^30^, and EfficientNet-B0^31^ were pre-trained using ImageNet to perform transfer learning. These networks were trained on the training dataset and evaluated using the test dataset to determine the best-performing model. These methods were implemented using Python 3.6, TensorFlow 1.15, and Keras 2.1. They were then evaluated in an environment with Windows 10 OS and an NVIDIA GeForce GTX 1080 Ti GPU. To train the models, the maximum number of epochs was set to 50. The batch size was set to 16 using the adaptive moment estimation (Adam) optimizer. Categorical cross-entropy was used as a loss function for multi-class problems.

### Evaluation methods

The k-fold cross-validation test can evaluate the generalization performance accurately. As for an unbiased estimate of the performance of the proposed method, we performed a fivefold cross-validation test to train the model and another to validate the model. The datasets were partitioned into five nearly equal-sized folds. Five iterations of training and validation were performed such that within each iteration, a different fold of the data was used for validation, whereas the remaining four folds were used for learning. Training and validation were iterated five times such that within each iteration, four folds were held out for training, and the remaining fold was used for validation. The results of the 5 analyses in a fivefold cross-validation were summed to create a confusion matrix.

Several metrics were used to evaluate the performance of the DCNN: recall, precision, F1 score, and accuracy. These metrics were calculated using a confusion matrix from true positives, true negatives, false positives, and false negatives.

## Data Availability

The DDSM dataset is available online at https://wiki.cancerimagingarchive.net/pages/viewpage.action?pageId=22516629.
